# Examining rheological behavior of CeO_2_-GO-SA/10W40 ternary hybrid nanofluid based on experiments and COMBI/ANN/RSM modeling

**DOI:** 10.1038/s41598-022-26253-4

**Published:** 2022-12-21

**Authors:** Mojtaba Sepehrnia, Hamid Maleki, Mahsa Karimi, Erfan Nabati

**Affiliations:** 1 Department of Mechanical Engineering, Shahabdanesh University, Qom, Iran; 2 Department of Mechanical Engineering, Technical and Vocational University, Qom, Iran; 3 Department of Mechanical Engineering, Isfahan University of Technology, Isfahan, Iran; 4 Faculty of Mechanical Engineering, University of Kashan, Kashan, Iran

**Keywords:** Mechanical engineering, Fluid dynamics, Engineering, Mathematics and computing

## Abstract

In this study, the rheological behavior and dynamic viscosity of 10W40 engine oil in the presence of ternary-hybrid nanomaterials of cerium oxide (CeO_2_), graphene oxide (GO), and silica aerogel (SA) were investigated experimentally. Nanofluid viscosity was measured over a volume fraction range of *VF* = 0.25–1.5%, a temperature range of *T* = 5–55 °C, and a shear rate range of *SR* = 40–1000 rpm. The preparation of ternary-hybrid nanofluids involved a two-step process, and the nanomaterials were dispersed in SAE 10W40 using a magnetic stirrer and ultrasonic device. In addition, CeO_2_, GO, and SA nanoadditives underwent X-ray diffraction-based structural analysis. The non-Newtonian (pseudoplastic) behavior of ternary-hybrid nanofluid at all temperatures and volume fractions is revealed by analyzing shear stress, dynamic viscosity, and power-law model coefficients. However, the nanofluids tend to Newtonian behavior at low temperatures. For instance, dynamic viscosity declines with increasing shear rate between 4.51% (at 5 °C) and 41.59% (at 55 °C) for the 1.5 vol% nanofluid. The experimental results demonstrated that the viscosity of ternary-hybrid nanofluid declines with increasing temperature and decreasing volume fraction. For instance, assuming a constant SR of 100 rpm and a temperature increase from 5 to 55 °C, the dynamic viscosity increases by at least 95.05% (base fluid) and no more than 95.82% (1.5 vol% nanofluid). Furthermore, by increasing the volume fraction from 0 to 1.5%, the dynamic viscosity increases by a minimum of 14.74% (at 5 °C) and a maximum of 35.94% (at 55 °C). Moreover, different methods (COMBI algorithm, GMDH-type ANN, and RSM) were used to develop models for the nanofluid's dynamic viscosity, and their accuracy and complexity were compared. The COMBI algorithm with R^2^ = 0.9995 had the highest accuracy among the developed models. Additionally, RSM and COMBI were able to generate predictive models with the least complexity.

## Introduction

In the twenty-first century, there have been many efforts to promote Sustainable Development Goals (SDGs) in the automotive industry^1^. The development of various components of vehicles has been followed in line with this goal. One of the most critical components of any vehicle is engine oil, which flows like blood through the engine's veins and lubricates moving parts, cools the engine, improves sealing, cleans the engine, and prevents corrosion. Dispersing nanomaterials with high thermal conductivity in engine oil is one of the most effective methods for removing excess heat from moving parts and increasing engine efficiency. Choi^2^ proposed the idea of dispersing various nano-sized particles, including metal^3^, metal oxide^4,5^, and carbon-based nanomaterials^6^, for the first time in 1995 to improve the properties of the base fluid. Following the successful implementation of this concept, researchers conducted numerical^7^, analytical^8^, and experimental^9,10^ studies in various fields using nanofluids.

Although using nanomaterials can significantly improve the thermal properties of the base fluid, the hydrodynamic properties can be adversely affected^11^. Among these properties, the viscosity of the nanofluid, as an influential parameter in calculating the pumping power, can be a turning point in the application of nanoparticle (NP)^12^ concentration and type. In addition, the amount of viscosity influences the Rayleigh and Reynolds numbers, which play a crucial role in determining convective heat transfer. On the other hand, it must be noted that increasing the dynamic viscosity, particularly at higher engine oil temperatures, is advantageous in terms of lubrication^13^.

In recent years, hybrid nanofluids have attracted the interest of numerous researchers as a new class of nanofluids^14–18^. These nanofluids are made by combining two or more distinct nanomaterials with a base fluid. Although research on hybrid nanofluids is not as extensive as on mono nanofluids, preliminary research indicates that binary and ternary-hybrid nanofluids can improve thermal and chemical properties while preventing excessive effective viscosity^19^. In terms of research on the dynamic viscosity of hybrid nanofluids, binary-hybrid nanofluids have received far more attention than ternary-hybrid nanofluids.

To this end, Soltani and Akbari^20^ experimented to determine the effects of nano-sized concentration and temperature on MgO-MWCNT/ethylene glycol binary hybrid nanofluid. In their study, the system temperature and nanoparticle loadings were between 30 and 60 °C and 0 and 1%, respectively. Their findings demonstrated that MgO-MWCNT/ethylene glycol behaves as a Newtonian fluid under the investigated conditions. In addition, they observed that increasing the nanoparticle concentration from 0.1 to 1% increased the dynamic viscosity by 168%.

Zareie and Akbari^21^ examined the rheological behavior of MgO-MWCNTs/water-EG nanofluids in a separate experimental study on binary nanofluids. They determined the viscosity at various shear rates (20–60 rpm), temperatures (25–60 °C), and NPs volume fractions (0.025–0.8%). According to their findings, increasing the nanoparticles concentration increases viscosity, whereas increasing the nanofluid temperature decreases viscosity. They also claimed that the nanofluids exhibited Newtonian behavior in every case.

Aghaei et al.^13^ measured the dynamic viscosity of CuO–MWCNTs/SAE 5w50 nanofluid using a Brookfield viscometer at temperatures ranging from 5 to 55 °C and NPs concentrations between 0.05 and 1%. At a temperature of 55 °C, the dispersion of 1% CuO–MWCNTs Nps increased the dynamic viscosity by 35.52%. However, adding a similar concentration of Nps at 15 °C increased dynamic viscosity by 12.92%.

Esfe et al.^22^ investigated the rheological behavior of MWCNT–TiO_2_/SAE50 nanofluid at temperatures between 25 and 50 °C and NPs concentrations between 0 and 1%. In all cases, the observation of shear stress and its relationship with shear rate revealed the nanofluid's non-Newtonian behavior and pseudoplasticity.

Asadi et al.^23^ determined that the shear rate, temperature, and NPs concentration influence the rheological behavior of the CuO–TiO_2_/water hybrid nanofluid. The shear stress analysis revealed that all nanofluid samples are of Newtonian type. The highest dynamic viscosity was recorded at 25 °C (the lowest temperature) and a concentration of 1 vol% NPs. Based on a cost–benefit analysis, they also concluded that the introduced nanofluid could be a superior alternative to pure water.

Said et al.^24^ conducted numerous tests on the thermophysical properties of a novel binary nanofluid (rGO–Co_3_O_4_/water) in the temperature range of 20–60 °C with NPs loadings ranging from 0.05 to 0.2 vol%. They reported a 70.83% increase in dynamic viscosity at 60 °C and a 0.2% increase in the volume fraction of NPs relative to the base fluid.

Zhu et al.^25^ investigated the effects of system temperature (25–50 °C) and NPs concentrations (0.1–0.6 vol%) on the dynamic viscosity of a water-Ethylene glycol (80:20) mixture fluid containing MWCNT–WO_3_ binary nanoparticles. Giwa et al.^26^ evaluated the dynamic viscosity of a binary nanofluid composed of MWCNT-Fe_2_O_3_ (20:80). Their results demonstrated that at 15 °C with NPs loadings of 1.5 vol%, the maximum increase in dynamic viscosity of nanofluid relative to base fluid is 35.7%. They also observed that binary nanofluids of MWCNT–Fe_2_O_3_/DIW had a lower viscosity than mono nanofluids of Fe_2_O_3_/DIW, which could significantly impact pumping power reduction.

In line with previous studies, Sepehrnia et al.^27^ recently conducted tests on 5W30 engine oil as the base fluid and ZnO–MWCNT (30:70) nanoparticles with a volume fraction in the range of 0.05 to 1 vol%. In all cases, the non-Newtonian (pseudoplastic) behavior of the hybrid nanofluid was observed at temperatures ranging from 5 to 55 °C and shear rates ranging from 50 to 1000 rpm. At elevated temperatures, the viscosity improvement of the hybrid nanofluid was considerably less than that of the base fluid.

Sajeeb and Rajendrakumar^28^ analyzed the rheological behavior of hybrid CeO_2_/CuO–coconut oil nanolubricants with 75/25, 50/50, and 25/75 CuO and CeO_2_ proportions. They observed that the nanofluid exhibited non-Newtonian behavior at all CuO:CeO_2_ ratios where the shear rates were low, and the NPs concentration was high. In addition, they claimed that by increasing the shear rate, the nanofluid behaved as a Newtonian fluid, regardless of the NP’s concentration and temperature.

Yadav et al.^29^ examined the rheological characteristics of CeO_2_–Al_2_O_3_(50:50)/EG, Al_2_O_3_/EG, and CeO_2_/EG nanofluids. They observed that mono nanofluids exhibit non-Newtonian (dilatant) behavior at elevated temperatures. Their findings also demonstrated that the hybrid nanofluid's hydrodynamic behavior is very similar to that of the base fluid, making it an excellent choice for anti-freezing applications.

As previously stated, the use of ternary nanomaterials has the potential to alter the properties of the base fluid significantly. Clearly, the ratio, type, and size of NPs significantly impact the thermal and rheological properties of the ternary-hybrid nanofluids. However, measuring the dynamic viscosity and describing the rheological behavior of ternary-hybrid nanofluids has received limited attention.

In one of these studies, Sahoo and Kumar^30^ examined the dynamic viscosity of a water-based Al_2_O_3_-CuO-TiO_2_ ternary-hybrid nanofluid at temperatures between 35 and 50 °C. The particle loadings of various samples ranged between 0.01 and 0.1%. The results of comparing mono, binary, and ternary nanofluids were intriguing. At the same volume fraction and temperature, mono nanofluid (CuO/water) exhibited the greatest dynamic viscosity, whereas binary nanofluid (Al_2_O_3_–TiO_2_/water) exhibited the least. The ternary hybrid nanofluid demonstrated a lower dynamic viscosity than CuO/water, but its performance was inferior to that of the binary nanofluids (Al_2_O_3_–TiO_2_/water and Al_2_O_3_–CuO/water). Afterward, Sahoo^31^ repeated the experiments with a different type of ternary-hybrid water-based nanofluid (Al_2_O_3_–SiC–TiO_2_/water). The percentage of each nanomaterial in the volume fraction of NPs was deemed to be equivalent. The findings demonstrated that increasing the concentration of NPs emphasizes the importance of internal resistance in ternary-hybrid nanofluids. In addition, it was observed that nanofluids with a low concentration of NPs exhibit a weak dynamic viscosity.

Dezfulizadeh et al.^32^ examined shear stress and dynamic viscosity for Cu–SiO_2_–MWCNT/water nanofluid and analyzed the effects of temperature (15–65 °C) and NPs concentration (1–3 vol%) on the target variables in a separate study on ternary-hybrid nanofluids. According to their results, the ternary-hybrid nanofluid displayed Newtonian behavior.

The thermophysical and rheological properties of water-based CuO/MgO/TiO_2_ ternary-hybrid nanofluids were investigated by Mousavi et al.^33^. They considered five different ratios for mixing nanomaterials at a volume concentration range of 0.1–0.5%. They examined the system at temperatures ranging from 15 to 60 °C.

In a separate study, Said et al.^34^ synthesized a novel ternary nanopowder (rGO–Fe_3_O_4_–TiO_2_), dispersed it in ethylene glycol as the base fluid, and then evaluated the nanofluid's stability, density, and viscosity. The various cases of their experiments included changes in temperature (25–50 °C) and NPs weight percent (0.01–0.25).

Recently, Zayan et al.^35^ analyzed the rheological properties of two ternary-hybrid nanofluids, GO–TiO_2_–Ag/water, and rGO–TiO_2_–Ag/water. The findings indicated that the viscosity of GO–TiO_2_–Ag and rGO–TiO_2_–Ag nanofluids increased by 40 and 33%, respectively, when the temperature and shear rate was increased. In all instances where the NPs concentrations were low, and the shear rates were high, the nanofluid exhibited non-Newtonian behavior, highlighting the importance of precise viscosity analysis for ternary-hybrid nanofluids.

In recent years, the application of machine learning (ML) approaches in modeling various phenomena has opened new horizons in practical fields^36,37^. Using experimental methods to predict nanofluids' rheological behavior and hydrodynamic properties is extremely time- and cost-intensive. One of the most popular methods for estimating the viscosity of hybrid nanofluids has been modeling through strategies based on soft computing^38,39^. Table 1 summarizes the application of soft computing based methods in modeling the dynamic viscosity of engine oil-based hybrid nanofluids for studies conducted between 2016 and the present. According to Table 1, numerous classical and ML methods, including artificial neural network (ANN), self-organizing map neural network (SOM-NN), group method of data handling neural network (GMDH-NN), multivariate linear regression (MLR), gene expression programming (GEP), multigene genetic programming (MGGP), response surface methodology (RSM), and least-squares support vector machines (LSSVM) have been used to predict the dynamic viscosity of engine oil-based hybrid nanofluids. Among these methods, ANN is the most prevalent used for viscosity modeling.

**Table 1:** Overview of the papers on machine learning approaches in dynamic viscosity modeling of engine oil-based hybrid nanofluids.

References	Base fluid	Nanomaterials	Independent variables	Method
Afrand et al.^40^	SAE50	MWCNT–SiO_2_	$T, \varphi$	ANN
Alirezaie et al.^41^	SAE40	MWCNT–MgO	$T, \varphi , \dot{ \gamma }$	ANN
Hemmat Esfe et al.^42^	10W40	MWCNT–TiO_2_	$T, \varphi , \dot{ \gamma }$	ANN
Hemmat Esfe et al.^43^	5W50	MWCNT–Al_2_O_3_	$T, \varphi$	ANN
Maddah et al.^44^	SAE 10W40, SAE 85W90	MWCNT–carbon	$T, \varphi , \dot{\gamma }$	SOM-NN
Nadooshan et al.^45^	10W40	MWCNT–SiO_2_	$T, \varphi , \dot{ \gamma }$	ANN
Hemmat Esfe et al.^46^	10W40	MWCNT–Al_2_O_3_	$T, \varphi , \dot{\gamma }$	ANN
Hemmat Esfe et al.^47^	5W50	MWCNT–Al_2_O_3_	$T, \varphi , \dot{\gamma }$	ANN
Hemmat Esfe et al.^48^	5W50	MWCNT–SiO_2_	$T, \varphi , \dot{ \gamma }$	ANN
Aghaei et al.^13^	SAE 5W50	MWCNT–CuO	$T, \varphi$	ANN
Hemmat Esfe et al.^49^	SAE50	MWCNT–Al_2_O_3_	$T, \varphi , \dot{\gamma }$	RSM
Sepehrnia et al.^27^	5W30	MWCNTs–ZnO	$T, \varphi , \dot{\gamma }$	ANN
Hemmat Esfe et al.^50^	5W50	MWCNT–Al_2_O_3_	$T, \varphi , \dot{\gamma }$	RSM
Hemmat Esfe et al.^51^	SAE50, SAE40, 5W50	MWCNT–ZnO	$T, \varphi$	RSM
Asadi et al.^52^	Engine oil	MWCNT–MgO	$T, \varphi , \dot{\gamma }$	LSSVM
Toghraie et al.^53^	Engine oil	MWCNT–WO_3_	$T, \varphi , \dot{ \gamma }$	ANN
Chu et al.^54^	5W40	MWCNT–TiO_2_	$T, \varphi , \dot{ \gamma }$	RSM, ANN
Hemmat Esfe et al.^55^	SAE 40	MWCNT–Al_2_O_3_	$T, \varphi$	ANN
Sepehrnia et al.^56^	5W30	MWCNT–SiO_2_	$T, \varphi , \dot{\gamma }$	GMDH-NN
Jamei and Ahmadianfar^57^	Various Engine oils	Various binary NPs	$T, \varphi , \rho , {D}_{p}$	MGGP, GEP, MLR

The findings presented in Table 1 reveal that methods such as ANN, SOM-NN, and LSSVM can improve the accuracy of dynamic viscosity prediction. Using optimized ANN architecture, Hemmat Esfe et al.^47^ and Aghaei et al.^13^ reported an R^2^ value of 0.9998 as a crucial statistical criterion. In addition, utilizing LSSVM and SOM-NN methods, Asadi et al.^52^ and Maddah et al.^44^ reported a correlation coefficient (R = 0.9999) that provides a highly desirable model. However, empirical correlation cannot be established using the methods above, which is deemed a limitation. It is possible to present a mathematical relationship between independent and dependent variables using methods such as GMDH, RSM, MLR, GEP, and MGGP. Indeed it must be acknowledged that these techniques typically produce less accurate results.

Esfe et al.^50,51^ reported an R^2^ = 0.9948 and 0.9996 via the RSM method. Furthermore, Chu et al.^54^ compared ANN and RSM techniques. The ANN method was more accurate than the RSM method (R^2^ = 0.999 vs. R^2^ = 0.991). The maximum error for the ANN method was 5%, whereas, for 38.2% of the datasets, the RSM method produced models with errors in the 5–10% range.

Sepehrnia et al.^56^ presented an accurate model with an R^2^ > 0.999 through the ANN model (GMDH-type). Moreover, a comparison of the models proposed by Jamei and Ahmadianfar^57^ indicated that approaches based on genetic programming were more capable of modeling the dynamic viscosity in terms of the various nanoparticle parameters.

A literature review reveals that ternary hybrid nanofluids have the potential to improve the properties of nanofluids significantly. In contrast, a few researchers have briefly considered studying this type of nanofluid. In this study, the rheological behavior and dynamic viscosity of 10W40 engine oil in the presence of ternary nanomaterials of cerium oxide (CeO_2_), graphene oxide (GO), and silica aerogel (SA) are investigated for the first time. The dynamic viscosity of engine oil-based ternary-hybrid nanofluids is measured over a broad range of nanoparticle volume fractions (0.25–1.5%), shear rates (40–1000 rpm), and temperatures (5–55 °C). Eventually, utilizing the experimental measurement datasets, three models for accurately predicting nanofluid viscosity are developed based on soft computing methods (COMBI algorithm, GMDH-type ANN, and RSM).

## Experiments

This section describes the properties of 10W40 engine oil as the base fluid and CeO_2_, GO, and SA as additive nanomaterials. In addition, the techniques and instruments required for characterization, preparation, and measurement of ternary-hybrid nanofluid rheological behavior and viscosity are discussed.

### Base fluid and nanomaterials

**Figure 1:**
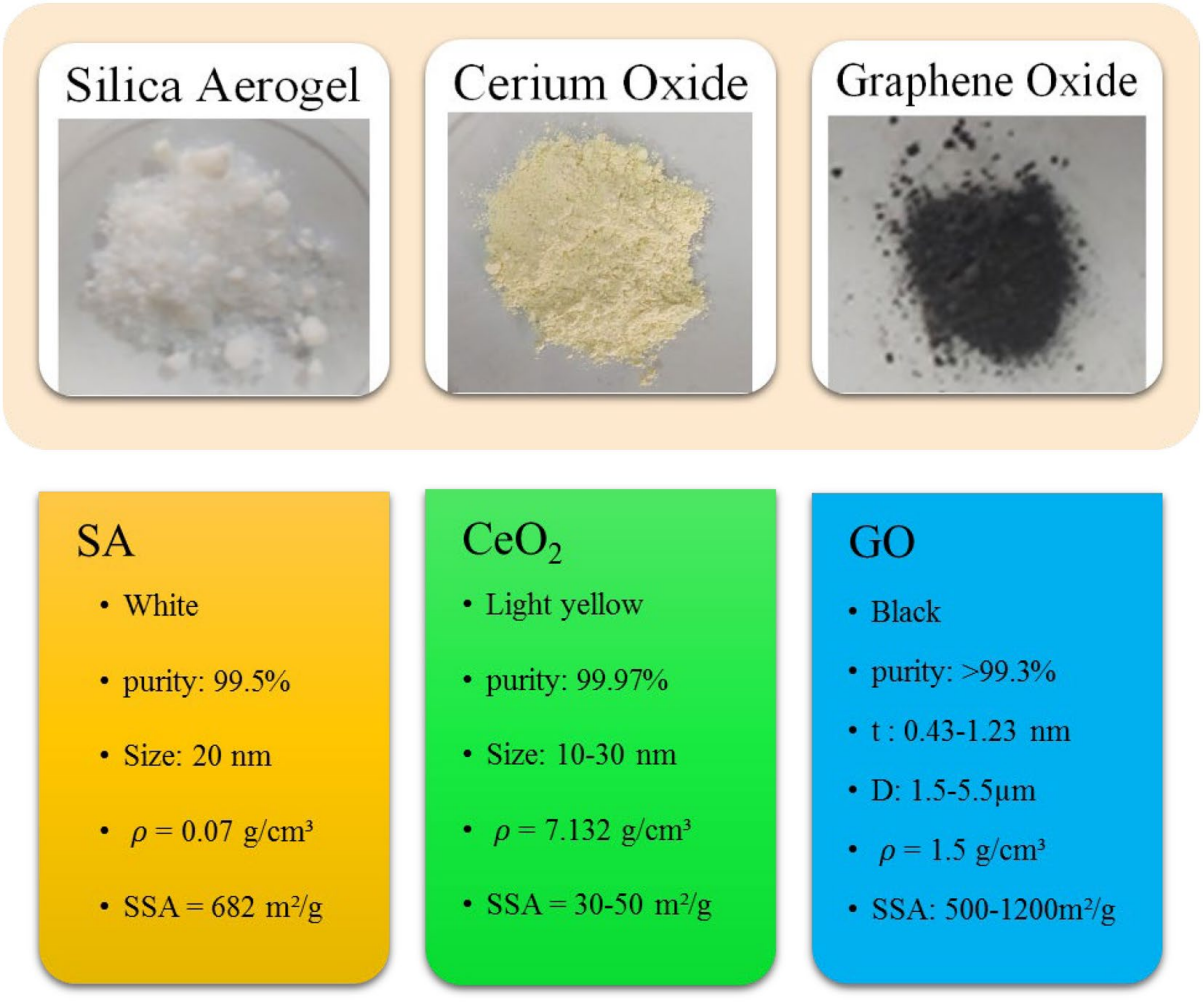
The characteristics of the used nanomaterials.

In our experiments, 10W40 engine oil (manufactured by Behran oil) served as the base fluid. The same proportion of ternary nanomaterials, cerium oxide (CeO_2_), graphene oxide (GO), and silica aerogel (SA), was dispersed in the base fluid. The characteristics of the nanomaterials are depicted in Fig. 1. The effect of using graphene oxide^58,59^ and cerium oxide^60,61^ nanoparticles in a fluid has been investigated in previous studies and it has been proven that it improves the thermal conductivity coefficient and increases the dynamic viscosity of the nanofluid. High specific surface area, high porosity, low density and low thermal conductivity are among the unique features of silica aerogel^62^. The low thermal conductivity of silica aerogel is one of its weak points, but the high porosity of silica aerogel makes the nanofluid stable, so that the general properties of the nanofluid are strengthened by adding graphene oxide and cerium oxide nanoparticles.

**Figure 2:**
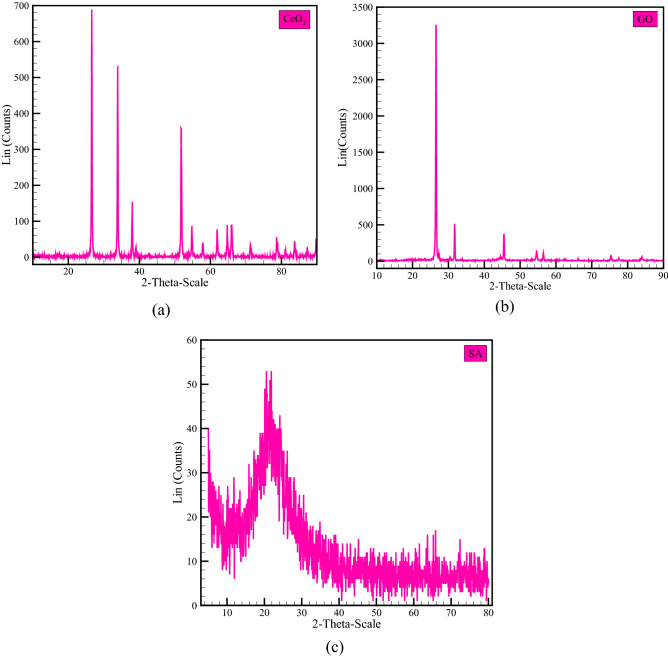
XRD graph: (**a**) cerium oxide, (**b**) graphene oxide and (**c**) silica aerogel.

X-ray diffraction (XRD) is an old but widely used analytical technique for determining atomic distance, crystal structure, and material purity. In fact, this non-destructive technique is one of the most important tools for providing various information on the scale of cell dimensions^63^. Figure 2 depicts the structural analysis performed by the XRD method on CeO_2_, GO, and SA nanoadditives. The intensity of the XRD peaks reveals that the nanomaterials under investigation have formed a suitable crystalline phase structure. Furthermore, the peaks that indicate impurity are not observed in the XRD report, implying that the GO, CeO_2_, and SA powders have the appropriate ability to produce a single phase.

### Ternary-hybrid nanofluid preparation

The 10W40 engine oil-based GO/CeO_2_/SA ternary-hybrid nanofluid was analyzed utilizing six different nanoparticle volume fractions ($\varphi =0.25\%, 0.5\%, 0.75\%, 1\%, 1.25\%, 1.5\%$). If in this type of special composition, the volume fraction of nanoparticles is more than 1.5%, the instability of nanoparticles in the base fluid and adhesion of nanoparticles are observed in less than a few hours. Therefore, nanofluids were not prepared for a volume fraction greater than 1.5%. The relationship below was employed to prepare ternary-hybrid nanofluids in various VFs:

1 $$\varphi =\left[\frac{{\left(\frac{M}{\rho }\right)}_{Ce{O}_{2}}+{\left(\frac{M}{\rho }\right)}_{GO}+{\left(\frac{M}{\rho }\right)}_{SA}}{{\left(\frac{M}{\rho }\right)}_{Ce{O}_{2}}+{\left(\frac{M}{\rho }\right)}_{GO}+{\left(\frac{M}{\rho }\right)}_{SA}+{\left(\frac{M}{\rho }\right)}_{SAE10W40}}\right]\times 100$$

where $M$ and $\rho$ denote mass and density, respectively. An electronic balance (model: AND 600 GF) with a 1 mg accuracy was used to calculate the mass percentage of each nanomaterial for different $\varphi$.

**Figure 3:**
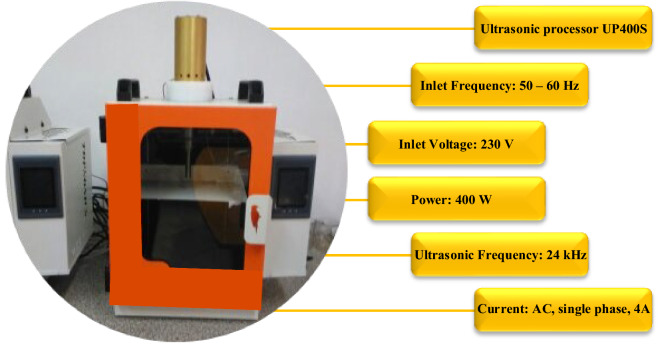
The ultrasonic bath device and ultrasonic processor specifications.

**Figure 4:**
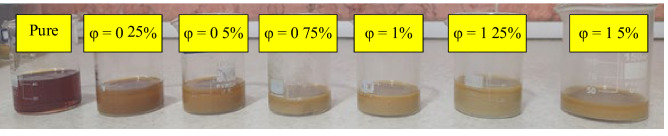
Ternary-hybrid nanofluids samples in different VFs.

Standard techniques for preparing nanofluids include one-step and two-step methods. In the one-step method, preparation and dispersion of nanoparticles in the base fluid co-occur, whereas, in the two-step method, preparation is the first step, followed by dispersion by ultrasonic irradiation or mechanical stirring in the second step. The two-step method has the benefits of being simple, inexpensive, and more compatible with oxide nanomaterials. The present study prepared ternary-hybrid nanofluids in two steps due to the stated advantages. Initially, a magnetic stirrer was utilized for 1 h to mix the solution during preparation. As depicted in Fig. 3, the ultrasonic process was performed for 2 h to prevent the accumulation and adhesion of nanomaterials. The amount of time to use the magnetic stirrer and ultrasonic device is determined according to the number and type of nanoparticles and user's experience. Figure 4 depicts samples of prepared nanofluids with different volume fractions.

### Viscosity measurement

**Figure 5:**
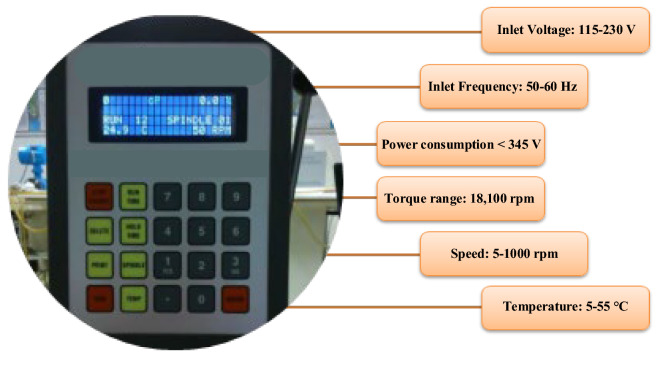
Brookfield viscometer model CAP2000+ and its technical specifications.

In the present experiments, the dynamic viscosity of nanofluids was measured using a Brookfield CAP2000+ viscometer. Figure 5 depicts the image and technical specifications of the device. The calibration process was performed at room temperature using a base fluid to increase precision. Additionally, each experiment was repeated twice to reduce measurement error, and its mean was recorded on the datasheet. Table 2 presents the studied temperature, volume fraction, and shear rate ranges. The selection of temperature and shear rate ranges based on the measuring limit of the viscometer device and similar to the papers^64–66^. The selection of volume fraction depends on adhesion and accumulation of nanoparticles; in the present study, if volume fraction is selected more than 1.5%, accumulation of nanoparticles occurs.

**Table 2:** The studied range for input variables.

Input variables	From	To	Selected values
Temperature (°C)	5	55	5, 15, 25, 35, 45, 55
NPs volume fraction (%)	0	1.5	0, 0.25, 0.5, 0.75, 1, 1.25, 1.5
Shear rate (rpm)	40	1000	40, 60, 80, 100, 150, 200, 250, 300, 400, 500, 600, 700, 800, 900, 1000

## Results and discussion

### Rheological behavior

The rheological behavior of fluids is crucial in a variety of industrial applications such as nano-lubricant^67,68^ and nano-antifreeze^69^. This significant feature is determined by analyzing the relation between shear rate and shear stress. Fluids are classified as either Newtonian or non-Newtonian based on their rheological behavior. The dynamic viscosity remains constant as the shear rate varies in Newtonian fluids. In contrast, the dynamic viscosity of non-Newtonian fluids varies significantly with a change in shear rate.

**Figure 6:**
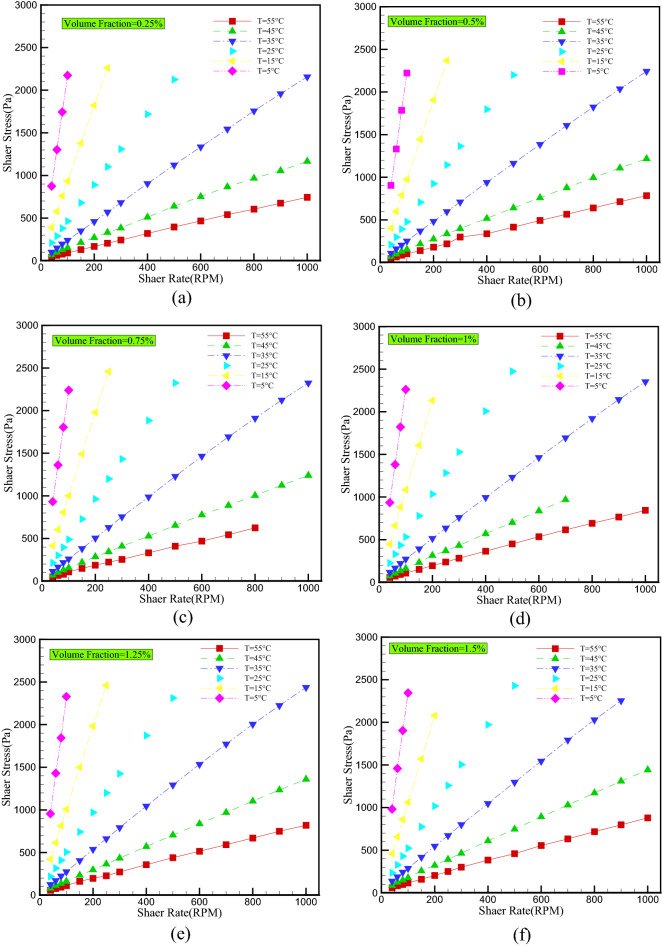
Shear stress versus shear rate for different temperature and VFs of (**a**) 0.25%, (**b**) 0.5%, (**c**) 0.75%, (**d**) 1%, (**e**) 1.25%, (**f**) 1.5%.

Figure 6 shows variations in shear stress as a function of shear rate at different temperatures and volume fractions. It is evident that shear stress increases with increasing shear rate in all volume fractions. Also, regardless of the shear rate, as the temperature rises, the cohesive force between molecules decreases, resulting in a reduction in shear stress. In addition, the slope of the shear stress diagram, which represents the nanofluid's viscosity, decreases as the shear rate increases, demonstrating the non-Newtonian behavior of the ternary-hybrid nanofluid. This change in the slope of the curve becomes more pronounced as the temperature rises. This indicates that the non-Newtonian behavior of nanofluids becomes more apparent as the temperature increases.

**Figure 7:**
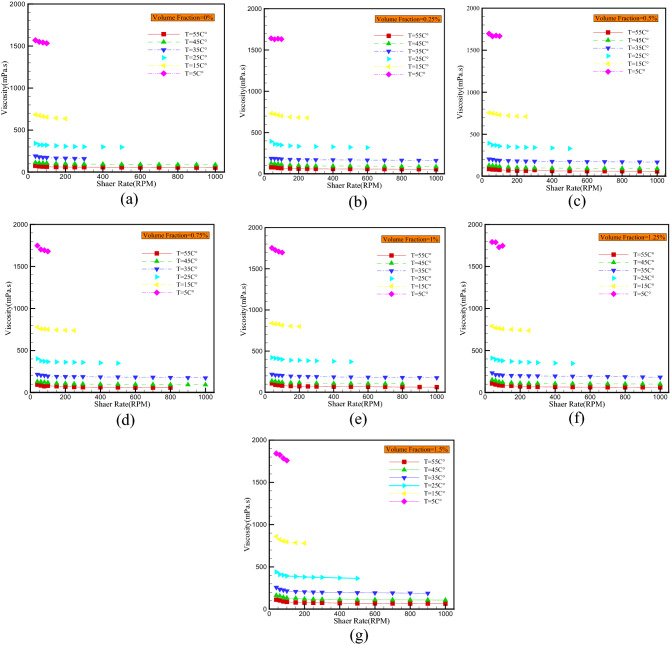
Dynamic viscosity versus shear rate for different temperature and VFs of (**a**) 0%, (**b**) 0.25%, (**c**) 0.5%, (**d**) 0.75%, (**e**) 1%, (**f**) 1.25%, (**g**) 1.5%.

In addition to shear stress, the analysis of viscosity curves in terms of shear rate can aid in determining the rheological behavior of nanofluids. In this regard, according to Fig. 7, viscosity decreases with increasing shear rate for all studied volume fractions, confirming the non-Newtonian behavior of the nanofluid. For instance, dynamic viscosity declines with increasing shear rate between 2.29% (at T = 5 °C) and 29.2% (at T = 55 °C) for the base fluid and between 4.51% (at T = 5 °C) and 41.59% (at T = 55 °C) for the nanofluid with volume fraction of 1.5%.

In addition to Figs. 6 and 7, the power-law model (Eq. 2) can be used to confirm the non-Newtonian behavior of the present nanofluid to identify its rheological behavior.

2 $$\tau =m{\dot{\gamma }}^{n}$$

where $m$ and $n$ are the consistency index and power law index, respectively, obtained by curve fitting the laboratory data. The value of the power law index can be used to determine whether a nanofluid is Newtonian or non-Newtonian:

3 $$\begin{gathered} n = 1 \to {\text{Newtonian }} \hfill \\ n < 1 \to {\text{Pseudoplastic }}\left( {{\text{non}} - {\text{Newtonian}}} \right){ } \hfill \\ n > 1 \to {\text{Dilatant }}\left( {{\text{non}} - {\text{Newtonian}}} \right){ } \hfill \\ \end{gathered}$$

Table 3 displays the maximum percentage of viscosity change due to a change in shear rate, along with the power-law model coefficients for each volume fraction and temperature. The results of the table indicate that the ternary-hybrid nanofluid is pseudoplastic; however, in certain volume fractions, the nanofluid tends to be Newtonian, particularly as the temperature decreases. According to the analysis, the CeO_2_–GO–SA/SAE 10W40 nanofluid's behavior is non-Newtonian in general.

**Table 3:** The maximum percentage of viscosity changes by altering the shear rate and related power-law model coefficients.

T (°C)	*φ* (%)	Maximum change in μ (%)	Best fitted model ($\tau =m{\dot{\gamma }}^{n}$)	
			m	n
55	0	29.2	0.1271	0.9077
	0.25	30.25	0.1567	0.8892
	0.5	30.02	0.1788	0.8818
	0.75	37.87	0.2372	0.8457
	1	41.39	0.2322	0.8598
	1.25	43.06	0.2813	0.8355
	1.5	41.59	0.2787	0.8439
45	0	23.01	0.1929	0.9154
	0.25	31.96	0.2683	0.8815
	0.5	29.22	0.2748	0.8798
	0.75	29.01	0.275	0.8822
	1	25.25	0.2632	0.8954
	1.25	32.89	0.3214	0.8717
	1.5	34.02	0.3585	0.8679
35	0	19.27	0.2849	0.9321
	0.25	13.94	0.2503	0.9544
	0.5	17.52	0.2886	0.9425
	0.75	19.31	0.2966	0.9452
	1	19.06	0.3174	0.9383
	1.25	21.88	0.3187	0.9426
	1.5	27.17	0.4248	0.9107
25	0	13.16	0.4665	0.9479
	0.25	19.09	0.6025	0.9262
	0.5	16.14	0.566	0.9385
	0.75	13.47	0.5171	0.9549
	1	12.01	0.5734	0.9506
	1.25	15.49	0.6042	0.9363
	1.5	17.35	0.6417	0.9348
15	0	7.16	0.9259	0.9521
	0.25	7.15	0.9742	0.9548
	0.5	5.83	0.9495	0.964
	0.75	5.19	0.9115	0.9734
	1	4.68	1.0276	0.968
	1.25	6.58	1.2708	0.9297
	1.5	9.30	1.2218	0.9419
5	0	2.29	1.8327	0.9751
	0.25	0.73	1.6914	0.9949
	0.5	1.89	1.883	0.9828
	0.75	3.89	2.2705	0.9578
	1	3.19	2.1956	0.9641
	1.25	3.41	2.2438	0.9645
	1.5	4.51	2.5518	0.9487

### Temperature effects on the viscosity

**Figure 8:**
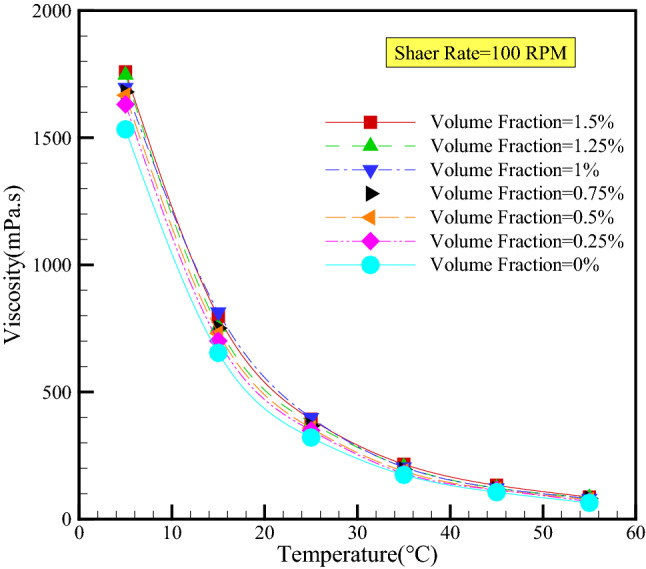
Effect of temperature on dynamic viscosity for various VFs in constant SR of 100 rpm.

It is evident that the fluid's temperature significantly impacts its viscosity to the point where it can play a significant role in the presence of nanomaterials and vice versa. Consequently, it is essential to investigate the effect of adding nanomaterials on the viscosity of fluids at various temperatures. Figure 8 depicts viscosity values in terms of temperature at a 100 rpm shear rate. As can be seen, the temperature is the most influential variable in changing viscosity, as an increase in temperature increases molecular motion and decreases van der Waals force and dynamic viscosity.

In addition, the viscosity of the nanofluid increases as the volume fraction rises. This is due to the increase in the intermolecular forces of nanomaterials and the increase in the interaction force between the molecules of SAE 10W40 and nanomaterials, which creates resistance to the movement of nanofluid and thus increases viscosity. It should be noted that as temperature rises, the effect of a rising volume fraction on viscosity increases, highlighting the importance of operating temperature when deciding whether or not to use nano-additives in various applications.

Quantitative analysis reveals that at a shear rate of 100 rpm and a temperature increase from 5 to 55 °C, the dynamic viscosity of the base fluid increases by at least 95.05% and no more than 95.82% (for the 1.5 vol% nanofluid). In addition, the dynamic viscosity increases by a minimum of 14.74% (at 5 °C) and a maximum of 35.94% (at 55 °C) when the volume fraction is increased from 0 to 1.5%.

### Effect of NPs' volume fraction on viscosity

**Figure 9:**
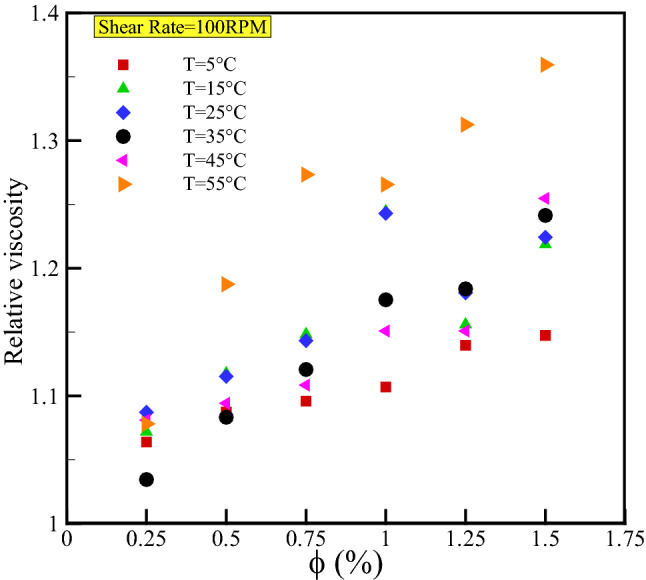
Effect of VF on relative viscosity for various temperatures in constant SR of 100 rpm.

Figure 9 depicts changes in relative viscosity (${\mu }_{r}={\mu }_{nf}/{\mu }_{bf}$) versus volume fractions for various temperatures to investigate the effect of NPs concentrations on nanofluid viscosity. In the presence of nanomaterials, it is evident that nanofluid viscosity is greater than that of the base fluid at all volume fractions. This is due to the increased interaction between nanofluid molecules compared to base fluid molecules. According to quantitative analysis depicted in Fig. 9, the most significant increase in relative viscosity occurs at a temperature of 55 °C and a volume fraction of 1.5%, which is equal to 94.35%; Conversely, the lowest relative viscosity increase of 3.45% is observed for nanofluid with a volume fraction of 0.25% and a temperature of 35 °C.

### Comparison of relative viscosity to established models

Various theoretical models have been proposed thus far to estimate the relative viscosity of nanofluids; the following models are among the most well-known:

Einstein^70^:

4 $${\mu }_{r}=1+2.5\varphi$$

Brinkman^71^:

5 $${\mu }_{r}={\left(1-\varphi \right)}^{-2.5}$$

Batchelor^72^:

6 $${\mu }_{r}=1+2.5\varphi +6.5{\varphi }^{2}$$

Wang et al.^73^:

7 $${\mu }_{r}=1+7.3\varphi +123{\varphi }^{2}$$

**Figure 10:**
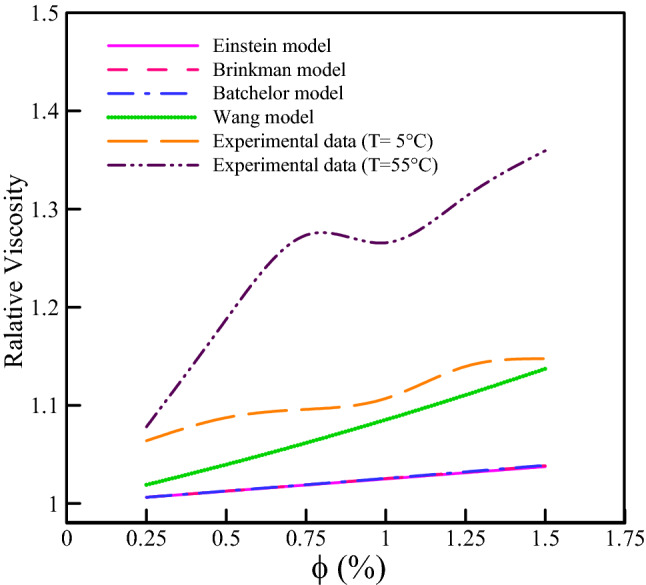
Comparison of our results with the various theoretical models.

Figure 10 compares the relative viscosity results of this study and the theoretical models. At two different temperatures, the theoretical models exhibit a linear behavior, whereas the results of the present study exhibit nonlinear behavior. Therefore, the viscosity of the present ternary-hybrid nanofluid cannot be estimated by the models previously cited. The following section develops different models based on RSM, GMDH-type ANN, and COMBI methods to accurately predict the present nanofluid's viscosity.

## Model development

### Response surface methodology (RSM)

The well-known RSM method attempts to establish a mathematical relationship between input and output variables by employing numerous mathematical models (linear, 2FI, quadratic, among others) and statistical criteria. Additionally, the RSM method can be used to determine optimal conditions. The RSM technique estimates model coefficients using the least-squares method.

The RSM analysis of the present laboratory data determined that the linear model provided adequate precision with less complexity than models with higher degrees. The variance analysis (ANOVA) and statistical parameters of the RSM's proposed model are presented in Tables 4 and 5. According to Table 4, the model and each of its terms have a p-value of less than 0.0001, demonstrating their significance. In addition, the F-value for the proposed model is 18,080.23, which demonstrates the model's validity. According to Table 5, Adeq Precision, which represents the signal-to-noise ratio, is significantly greater than the reasonable value (435.7319 >> 4), indicating that the proposed model is accurate. Equation (8) presents the dynamic viscosity correlation for the present ternary-hybrid nanofluid derived from the RSM method. The coefficient values of Eq. (8) are listed in Table 6.

**Table 4:** ANOVA-RSM linear model.

Source	Sum of squares	df	Mean square	F-Value	p-value Prob > F	
Model	1.45	3	0.4849	18,080.23	< 0.0001	Significant
A-T	0.0060	1	0.0060	224.26	< 0.0001	
B-phi	1.15	1	1.15	42,950.14	< 0.0001	
C-SR	0.0150	1	0.0150	560.81	< 0.0001	
Residual	0.0103	384	0.0000			
Cor Total	1.47	387

**Table 5:** Statistical parameters for proposed correlation by RSM.

Parameter	Value
Std. dev.	0.0052
C.V. %	1.26
Mean	0.4124
R-squared	0.9930
Adjusted R-Squared	0.9929
Predicted R-Squared	0.9928
Adeq precision	435.7319

**Table 6:** The coefficients of proposed correlation by RSM.

Parameter	Value
${\alpha }_{0}$	+ 0.27119
${\alpha }_{1}$	+ 3.90443E−003
${\alpha }_{2}$	− 9.18527E−003
${\alpha }_{3}$	+ 2.33542E−005

8 $${\left({\mu }_{nf}\right)}^{-0.17}={\alpha }_{0}+{\alpha }_{1}T+{\alpha }_{2}\varphi +{\alpha }_{3}\dot{\gamma }$$

The squared correlation coefficient (R^2^), which indicates the degree of similarity between laboratory data and the values predicted by the model, is one of the essential statistical parameters for evaluating proposed models. The closeness of its value to 1 indicates the proposed model's significant accuracy. If ${Y}_{i,Pred}$ is the predicted value and ${Y}_{i,Exp}$ is the experimental value of the *i*th dataset, R^2^ is defined as follows^74^:

9 $${R}^{2}=1-\sum_{i=1}^{n}\frac{{\left({Y}_{i,Pred}-{Y}_{i,Exp}\right)}^{2}}{{Y}_{i,Exp}^{2}}$$

According to Table 5, the values of R^2^, adjusted R^2^, and predicted R^2^ are respectively 0.9930, 0.9929, and 0.9928. The adjusted R^2^ considers the effect of the model's predicted constant coefficients, while the predicted R^2^ evaluates the model using datasets outside the range of laboratory data. Tables 4 and 5 demonstrate that the RSM's proposed model predicts the dynamic viscosity of the present ternary-hybrid nanofluid with high accuracy.

### GMDH-type neural network

Artificial neural networks are a computational model that, by imitating the function of neurons in the human brain, has become a potent tool for predicting and modeling complex phenomena. Nevertheless, the accuracy of ANN-based methods can significantly impact the quality and quantity of datasets. Ivakhnenko^75^ developed the group method of data handling (GMDH) polynomial neural networks based on feed-forward neural networks to maximize consistency in system behavior modeling and reduce dependence on the data structure. The self-organizing feature of the GMDH method is regarded as a significant advantage because, during the modeling process, only submodels that improve the final model's accuracy are retained. In recent years, the use of this method has increased significantly, particularly in research requiring the presentation of mathematical relationships between dependent and independent variables^76–78^.

To describe a system with $M$ datasets, a complex function such as $f$ is needed, which can connect inputs $x=\left({x}_{1},{x}_{2},\dots {x}_{n}\right)$ and output $y$:

10 $${y}_{i}=f\left({x}_{i1},{x}_{i2},\dots {x}_{in}\right) \quad (i=\mathrm{1,2},\dots ,M)$$

The objective of the GMDH method is to train a function such as $\widehat{f}$ so that the difference between predicted values of ${\widehat{y}}$ and real values of $y$ is minimized:

11 $${\widehat{y}}_{i}=\widehat{f}\left({x}_{i1},{x}_{i2},\dots {x}_{in}\right) \quad (i=\mathrm{1,2},\dots ,M)$$

12 $$\sum_{i=1}^{M}{\left[{\widehat{y}}_{i}-{y}_{i}\right]}^{2} \stackrel{ }{\to } min$$

To establish a connection between neurons (variables), it is possible to utilize different degrees of the Kolmogorov-Gabor polynomial^79^ with the following formula:

13 $$y={\beta }_{0}+\sum_{i=1}^{n}{\beta }_{i}{x}_{i}+\sum_{i=1}^{n}\sum_{j=1}^{n}{\beta }_{ij}{x}_{i}{x}_{j}+\sum_{i=1}^{n}\sum_{j=1}^{n}\sum_{k=1}^{n}{\beta }_{ijk}{x}_{i}{x}_{j}{x}_{k}+\dots$$

Previous analyses^79^ demonstrate that using the quadratic form of the Kolmogorov-Gabor polynomial strikes a fine balance between the model's complexity and accuracy:

14 $$y=G\left({x}_{i},{x}_{j}\right)={a}_{0}+{\beta }_{1}{x}_{i}+{\beta }_{2}{x}_{j}+{\beta }_{3}{x}_{i}{x}_{j}+{\beta }_{4}{x}_{i}^{2}+{\beta }_{5}{x}_{j}^{2}$$

**Figure 11:**
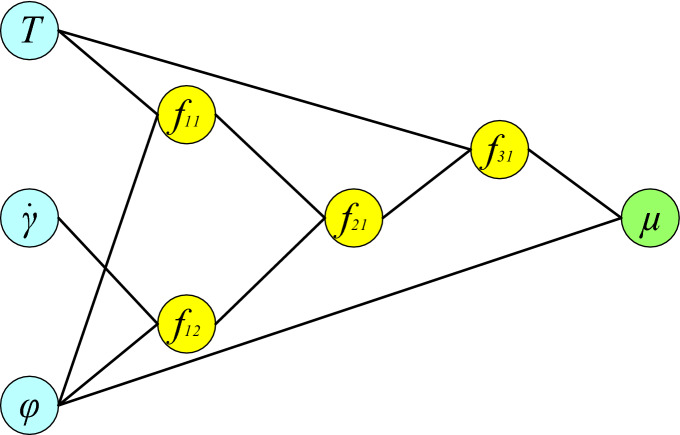
Structure of the developed GMDH-type ANN model.

It is also important to note that the least-squares method estimates model coefficients^80^. The available experimental data points are divided into two categories; the first category, comprising 80% of the data, is used to train the GMDH neural network, while the second category, comprising 20% of the data, is used to evaluate the resulting model. The structure of the obtained GMDH-type NN is shown in Fig. 11. This five-layer structure consists of three intermediate layers. The first layer contains the input variables (neurons), whereas the final layer holds the output variable. The connection between neurons is provided through the following relationships:

15 $${f}_{11}={\beta }_{\left(\mathrm{1,1}\right)}+{\beta }_{\left(\mathrm{1,2}\right)}T+{\beta }_{\left(\mathrm{1,3}\right)}\varphi +{\beta }_{\left(\mathrm{1,4}\right)}T\varphi +{\beta }_{\left(\mathrm{1,5}\right)}{T}^{2}+{\beta }_{\left(\mathrm{1,6}\right)}{\varphi }^{2}$$

16 $${f}_{12}={\beta }_{\left(\mathrm{2,1}\right)}+{\beta }_{\left(\mathrm{2,2}\right)}\dot{\gamma }+{\beta }_{\left(\mathrm{2,3}\right)}\varphi +{\beta }_{\left(\mathrm{2,4}\right)}\dot{\gamma }\varphi +{\beta }_{\left(\mathrm{2,5}\right)}{\dot{\gamma }}^{2}+{\beta }_{\left(\mathrm{2,6}\right)}{\varphi }^{2}$$

17 $${f}_{21}={\beta }_{\left(\mathrm{3,1}\right)}+{\beta }_{\left(\mathrm{3,2}\right)}{f}_{11}+{\beta }_{\left(\mathrm{3,3}\right)}{f}_{12}+{\beta }_{\left(\mathrm{3,4}\right)}{f}_{11}{f}_{12}+{\beta }_{\left(\mathrm{3,5}\right)}{f}_{11}^{2}+{\beta }_{\left(\mathrm{3,6}\right)}{f}_{12}^{2}$$

18 $${f}_{31}={\beta }_{\left(\mathrm{4,1}\right)}+{\beta }_{\left(\mathrm{4,2}\right)}{f}_{21}+{\beta }_{\left(\mathrm{4,3}\right)}T+{\beta }_{\left(\mathrm{4,4}\right)}{f}_{21}T+{\beta }_{\left(\mathrm{4,5}\right)}{f}_{21}^{2}+{\beta }_{\left(\mathrm{4,6}\right)}{T}^{2}$$

19 $${\mu }_{nf}={\beta }_{\left(\mathrm{5,1}\right)}+{\beta }_{\left(\mathrm{5,2}\right)}{f}_{31}+{\beta }_{\left(\mathrm{5,3}\right)}\varphi +{\beta }_{\left(\mathrm{5,4}\right)}{f}_{31}\varphi +{\beta }_{\left(\mathrm{5,5}\right)}{f}_{31}^{2}+{\beta }_{\left(\mathrm{5,6}\right)}{\varphi }^{2}$$

20 $${\beta }_{ij}=\left[\begin{array}{c}\begin{array}{ccc}\begin{array}{cc}1847.2700 & -80.591300\end{array} & \begin{array}{cc}98.352400& -1.4803600\end{array}& \begin{array}{cc}0.89085200& -7.58450 \end{array}\end{array}\\ \begin{array}{ccc}\begin{array}{cc}599.69600 & -1.532590 \end{array}& \begin{array}{cc}0.0000000 & 0.01250620 \end{array}& \begin{array}{cc}0.001091510& 0.0000000\end{array}\end{array}\\ \begin{array}{c}\begin{array}{ccc}\begin{array}{cc}77.0489 & 0.394016\end{array}& \begin{array}{cc}-0.111981& 7.97949\mathrm{e}-05\end{array}& \begin{array}{cc}0.000396126& 0.000309009\end{array}\end{array}\\ \begin{array}{ccc}\begin{array}{cc}-2.8747000& 0.77021000\end{array}& \begin{array}{cc}7.0523300& 0.000000\end{array}& \begin{array}{cc}0.000129245& -0.13827\end{array}\end{array}00\\ \begin{array}{ccc}\begin{array}{cc}-6.318800& 1.0138400\end{array}& \begin{array}{cc}0.0000000& -0.0190270\end{array}& \begin{array}{cc}1.62062\mathrm{e}-06& 7.2432500\end{array}\end{array}\end{array}\end{array}\right]$$

The R^2^ derived from the model provided by the GMDH method for the train and test datasets was 0.9993 and 0.9994, respectively, owing to the method's appropriate accuracy in predicting the dynamic viscosity of the studied nanofluid.

### Combinatorial (COMBI) algorithm

The combinatorial algorithm has a similar approach to the GMDH-type neural network, except that the COMBI algorithm employs a single-layer structure due to the complexity of the model-building procedure. The combinatorial algorithm used in the present study can be described in the following four steps^81^:

1. The following expression generates the sum of combinations of input variables (or their functions):
  21 $$y={\xi }_{0}+\sum_{i=1}^{n}{\xi }_{i}g\left({x}_{i}\right)+\sum_{i=1}^{n}\sum_{j=1}^{n}{\xi }_{ij}g\left({x}_{i}\right)\cdot g\left({x}_{j}\right)+\sum_{i=1}^{n}\sum_{j=1}^{n}{\xi }_{ij}g\left({x}_{i}\right)/g\left({x}_{j}\right)$$
  $g\left(x\right)$ can include various operators that are applied to input variables (${x}_{i}$), for example, functions such as square root, cube root, exponent, sigmoid, and trigonometric, among others.
2. Model coefficients are computed using the least-squares method at each neuron for training datasets.
3. Through the validation criterion (RMSE), the neuron errors are compared using testing datasets.
4. The final model is developed by combining optimal neurons with the least possible error and the maximum acceptable complexity.

The COMBI algorithm is a time-consuming method with a high calculation cost because it performs a thorough search between terms that improve the model and sometimes suggests models with a very high level of complexity. It is evident that the model's simplicity decreases its accuracy. Therefore, by limiting the model's complexity to 16 terms, the optimal model is obtained as follows:

22 $${\mu }_{nf}={\xi }_{0}+{\xi }_{1}\frac{1}{\sqrt{T}\sqrt[3]{\varphi }}+{\xi }_{2}{\mathrm{tan}}^{-1}\left(T\right){\mathrm{tan}}^{-1}\left(\dot{\gamma }\right)+{\xi }_{3}\frac{{\mathrm{tan}}^{-1}\left(T\right)}{{\mathrm{tan}}^{-1}\left(\dot{\gamma }\right)}+{\xi }_{4}\dot{\gamma }{\mathrm{tan}}^{-1}\left(T\right)+{\xi }_{5}\frac{{\mathrm{tan}}^{-1}\left(T\right)}{\dot{\gamma }}+{\xi }_{6}\frac{1}{{\mathrm{tan}}^{-1}\left(T\right)}+{\xi }_{7}\frac{T}{{\mathrm{tan}}^{-1}\left(T\right)}+{\xi }_{8}\frac{1}{T{\mathrm{tan}}^{-1}\left(T\right)}+{\xi }_{9}\frac{{\varphi }^{2}}{{\mathrm{tan}}^{-1}\left(T\right)}+{\xi }_{10}\frac{1}{{{\varphi }^{2}\mathrm{tan}}^{-1}\left(T\right)}+{\xi }_{11}\frac{\sqrt{\varphi }}{{\mathrm{tan}}^{-1}\left(T\right)}+{\xi }_{12}\frac{1}{{\sqrt{\varphi }\mathrm{ tan}}^{-1}\left(T\right)}+{\xi }_{13}\frac{{\varphi }^{3}}{{\mathrm{ tan}}^{-1}\left(T\right)}+{\xi }_{14}\frac{{\dot{\gamma }}^{2}}{{\mathrm{ tan}}^{-1}\left(T\right)}+{\xi }_{15}\frac{\mathrm{sin}\left(\varphi \right)}{\sqrt{\varphi }}$$

Like GMDH modeling, 80% of the data points were assigned to training and the rest to model testing. The coefficients of the model proposed by the COMBI algorithm are presented in Table 7. The value of R^2^ for both test and train data is equal to 0.9995. The high accuracy of the COMBI algorithm in the development of predictive models was reported in various fields^82–84^.

**Table 7:** The coefficients of proposed correlation by COMBI algorithm.

Parameter	Value
${\xi }_{0}$	1.96053e+06
${\xi }_{1}$	− 1395.76
${\xi }_{2}$	146,725
${\xi }_{3}$	− 733,260
${\xi }_{4}$	0.000638218
${\xi }_{5}$	445,799
${\xi }_{6}$	− 2.52395e+06
${\xi }_{7}$	7.52899
${\xi }_{8}$	1.27086e+06
${\xi }_{9}$	− 3884.6
${\xi }_{10}$	− 194.976
${\xi }_{11}$	15,097.7
${\xi }_{12}$	10,050.3
${\xi }_{13}$	1387.09
${\xi }_{14}$	388,568
${\xi }_{15}$	3153.95

### Accuracy and complexity of models

Two essential criteria can be used to evaluate the mathematical correlations provided for predicting diverse systems. The first, the most crucial, is to evaluate the model's accuracy using statistical parameters such as R^2^, RMSE, MAPE, and others. The second item is the degree of complexity, which indicates the predictive model's number of terms. The correlation between accuracy and complexity is typically positive in all modeling techniques. This section compares the complexity and precision of the presented models.

For a comprehensive comparison of the accuracy of the proposed models, the following two additional statistical parameters are introduced:

23 $$RMSE=\sqrt{\frac{1}{n}\sum_{i=1}^{n}{\left({Y}_{i,Pred}-{Y}_{i,Exp}\right)}^{2}}$$

24 $$MAPE=\left(\frac{1}{n}\sum_{i=1}^{n}\left|\frac{{Y}_{i,Pred}-{Y}_{i,Exp}}{{Y}_{i,Exp}}\right|\right)\times 100$$

**Figure 12:**
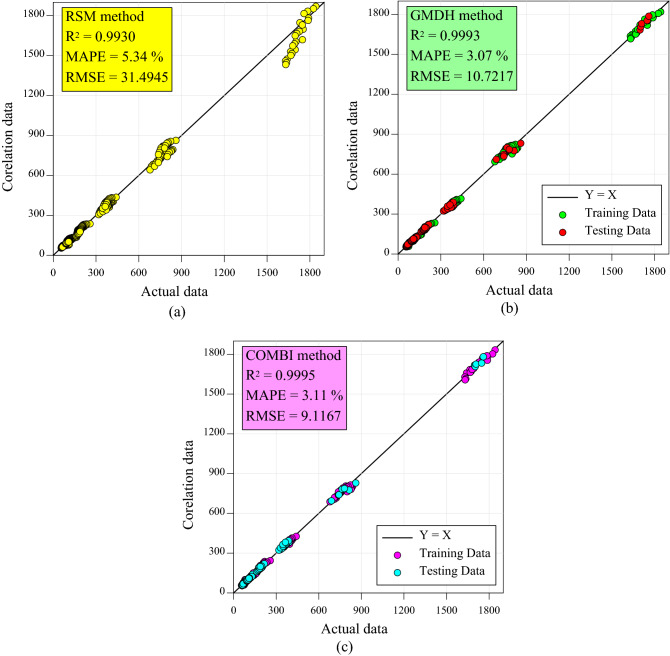
Comparison between experimental data with correlation outputs of (**a**) RSM model, (**b**) GMDH-type ANN model, and (**c**) COMBI algorithm.

Figure 12 compares the actual laboratory data values to those derived from the proposed models. As shown in the figure, the COMBI algorithm is more accurate at predicting the nanofluid's dynamic viscosity based on statistical criteria.

The accuracy and complexity of the proposed RSM, GMDH, and COMBI models are compared in Table 8. As can be seen, various models can provide different degrees of accuracy depending on the level of complexity. By reducing the complexity of the RSM model to four terms, reasonable accuracy is achieved. Meanwhile, GMDH-type ANN and COMBI algorithms offer more accurate models as their complexity increases.

**Table 8:** Comparison of the accuracy and complexity of the proposed models.

Criteria	Proposed models		
	RSM	GMDH-type ANN	COMBI algorithm
R^2^	0.9930	0.9993	0.9995
RMSE	31.4945	10.7217	9.1167
MAPE (%)	5.34	3.07	3.11
Complexity	4	30	16

It is possible to increase the complexity of all models to achieve greater accuracy. In the RSM method, for instance, R^2^ reaches 0.9995 when a fifth-order model with 56 different terms is considered. In addition, when a ten-layer structure is applied to the GMDH neural network, R^2^ equals 0.9995, indicating that the accuracy and complexity of the resulting model increase. Also, for the COMBI algorithm, a value of 0.9998 was observed for R^2^ in a case with model complexity equal to 80.

If model complexity is more important than accuracy, the robust COMBI algorithm can produce the following model with an R^2^ = 0.9992:

25 $${\mu }_{nf}=1576.36-7587.26\frac{{\mathrm{tan}}^{-1}\left(\dot{\gamma }\right)}{\sqrt[3]{T}}+22326.3\frac{\mathrm{ln}T}{T}-1639.96\frac{1}{T{e}^{\varphi }}$$

Additionally, it should be noted that all proposed models are only valid within the range of parameters that have been examined ($5^\circ{\rm C} \le T\le 55^\circ{\rm C} , \%0.25\le \varphi \le 1.5\%, 40 \mathrm{ rpm}\le \dot{\gamma }\le 1000 \mathrm{rpm}$). The proposed models can help to reduce the time and resources needed for experiments. Also, the models in the present study can be used in a variety of applications such as heat sinks^85–87^, heat pipes^88^, microchannels^89,90^, heat exchangers^91–93^, enclosures^94,95^, solar energy^9,96,97^ and automotive industry^98–101^.

## Uncertainty

In this section, the uncertainty analysis of the developed models is carried out to evaluate the dynamic viscosity prediction capability of the models. For this purpose, an approximately 95% confidence band around predicted values based on the Wilson score without continuity correction can be obtained using $\pm 1.96{S}_{de}$^102,103^. The mean error and standard deviation of prediction error are calculated as follows:

26 $$\overline{e }=\frac{1}{n}\sum_{i=1}^{n}\left({Y}_{i,Pred}-{Y}_{i,Exp}\right)$$

27 $${S}_{de}=\sqrt{\frac{\sum {\left({e}_{j}-\overline{e }\right)}^{2}}{n-1}}$$

where n denotes the number of data points.

This analysis is applied to all data for the RSM model and the test data points for the COMBI and GMDH models. The results of uncertainty analysis including mean error, standard deviation of prediction error, width of uncertainty band (WUB), and 95% interval of error prediction for different models are shown in Table 9. A positive value of $\overline{{\varvec{e}} }$ indicates an overestimation of the actual values and a negative value indicates an underestimation. According to Table 9, the model obtained from the COMBI algorithm shows the lowest uncertainty band (WUB = 35.2402), which indicates its significant reliability in providing accurate outputs compared to other models.

**Table 9:** Uncertainty analysis results for viscosity predictive models.

Model	$\overline{e }$	${S}_{de}$	WUB	95% interval of error prediction
RSM	− 4.5673	31.1599	122.1467	− 65.6407 to 56.5061
GMDH	1.20228	10.0239	39.2936	− 18.4445 to 20.8491
COMBI	0.7936	8.9898	35.2402	− 16.8265 to 18.4137

## Sensitivity analysis

The influence of independent variables on the responses of a system is checked utilizing sensitivity analysis. The significant reaction of a response to a slight change in an input variable indicates the high importance of that input. In the present research, the procedure by Esfe et al.^49,50,104,105^ is used for sensitivity analysis. For this purpose, the following equation is applied to calculate viscosity sensitivity:

28 $$Viscosity \; sensitivity (\%) =\left[\frac{{\left({\mu }_{nf}\right)}_{after \; change}}{{\left({\mu }_{nf}\right)}_{before \; change}}-1\right]\times 100$$

The viscosity sensitivity analysis of CeO_2_-GO-SA/10W40 ternary hybrid nanofluid is performed by applying a 10% change in VF for various temperatures. To augment the precision of the calculations, the most accurate developed model (COMBI) is applied.

**Figure 13:**
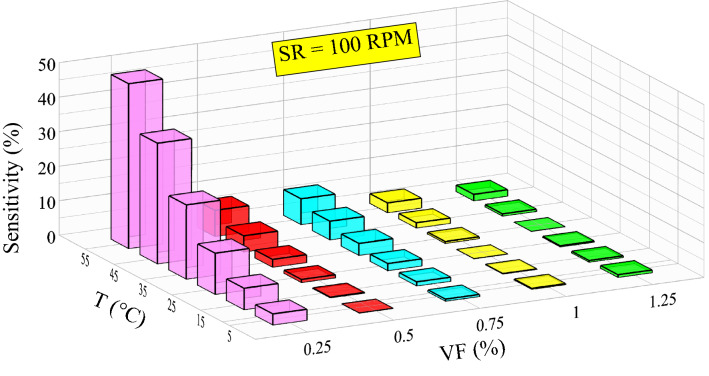
Viscosity sensitivity versus VF at different temperatures.

Figure 13 depicts the results of viscosity sensitivity analysis for the SR of 100 rpm. According to Fig. 13, with raising the temperature, viscosity sensitivity has an increasing trend for VFs < 0.75%. While for the VFs of 1% and 1.25%, with increasing temperature, the viscosity sensitivity first declines and then experiences a slight growth. Viscosity shows a high sensitivity to temperature variation in the lowest volume fraction (0.25%). The highest viscosity sensitivity occurs for the VF = 0.25% at temperature of 55 °C, which is equal to 47.8%.

The results deduced from the present sensitivity analysis are confirmation that double precision should be used in the preparation process of CeO_2_-GO-SA/10W40 ternary hybrid nanofluid, especially in low VFs and high temperatures, because possible errors in the production process strongly affect the viscosity and rheological behavior of the resulting nanofluid. Low viscosity sensitivity in VFs of 0.5–1.25% allows industries to use the nanofluid in special applications at different temperatures.

## Conclusion

In the present experimental study, the effects of temperature (5–55 °C), NP volume fraction (0.25–1.5%), and shear rate (40–1000 rpm) on the rheological behavior and dynamic viscosity of GO/CeO_2_/SA ternary-hybrid nanofluid based on 10W40 engine oil were analyzed. Moreover, different techniques (COMBI algorithm, GMDH-type ANN, and RSM) were utilized to develop models for the nanofluid's dynamic viscosity, and their accuracy and complexity were compared. The significant findings of this study can be stated as follows:

- Changes in shear stress, dynamic viscosity, and power-law model coefficients indicate that ternary-hybrid nanofluids exhibit non-Newtonian behavior at all temperatures and volume fractions. However, the nanofluids tend to Newtonian behavior at low temperatures.
- The values of the power law index ($n<1$) in all samples demonstrate that the nanofluid under study is pseudoplastic.
- The viscosity of ternary-hybrid nanofluid decreases with increasing temperature and shear rate or with decreasing nanomaterial volume fraction.
- Among the models developed to predict the dynamic viscosity of GO/CeO_2_/SA nanofluid based on 10W40 engine oil, the COMBI algorithm with R^2^ = 0.9995 provided the highest accuracy.
- The models obtained from the RSM and GMDH methods provides an accuracy equal to R^2^ = 0.9930 and R^2^ = 0.9993, respectively.
- Among the methods presented, the RSM method and the COMBI algorithm can produce predictive models with the least complexity.
- Ignoring the model's complexity, the COMBI algorithm can produce a model with R^2^ = 0.9998.
- Modeling with the COMBI algorithm is recommended because it has a high potential in developing models with high precision and low complexity.
- The sensitivity analysis for the present ternary hybrid nanofluid demonstrates that the viscosity sensitivity is maximized for VF of 0.25% at high temperatures.

## Data Availability

All data analyzed during this study are included in this published article.

## List of symbols

$$M$$: Mass (kg)
$$m$$: Consistency index (–)
$n$: Power law index (–)
$$T$$: Temperature (°C)

## Subscripts

bf: Base fluid
Exp: Experimental
nf: Nanofluid
Pred: Predicted
r: Relative

## Greek symbols

$\dot{\gamma }$: Shear rate (rpm)
$\mu$: Dynamic viscosity (cP)
$$\rho$$: Density (kg m^−^^3^)
$\varphi$: Nano-additives volume fraction (%)
$\tau$: Shear stress (Pa)

## Abbreviations

ANN: Artificial neural network
GMDH: Group method of data handling
MAPE: Mean absolute percentage error
NP: Nanoparticles
RMSE: Root mean squared error
RSM: Response Surface methodology
SAE: Society of automotive engineers
SR: Shear rate
VF: Volume fraction
XRD: X-Ray diffraction
